# Supplementary material for: The interactome of intact mitochondria by cross-linking mass spectrometry provides evidence for coexisting respiratory supercomplexes
Source: Mol Cell Proteomics. 2017 Dec 8;17(2):216–32. doi: 10.1074/mcp.RA117.000470 (PMC5795388; doi:10.1074/mcp.RA117.000470)
Supplement: Supplemental Data [file supp_RA117.000470_133922_0_supp_23978_fzffwf.zip › spectra_annotation/mito_DR_spectra_annotation/343-1-18-1-10-1.pdf]

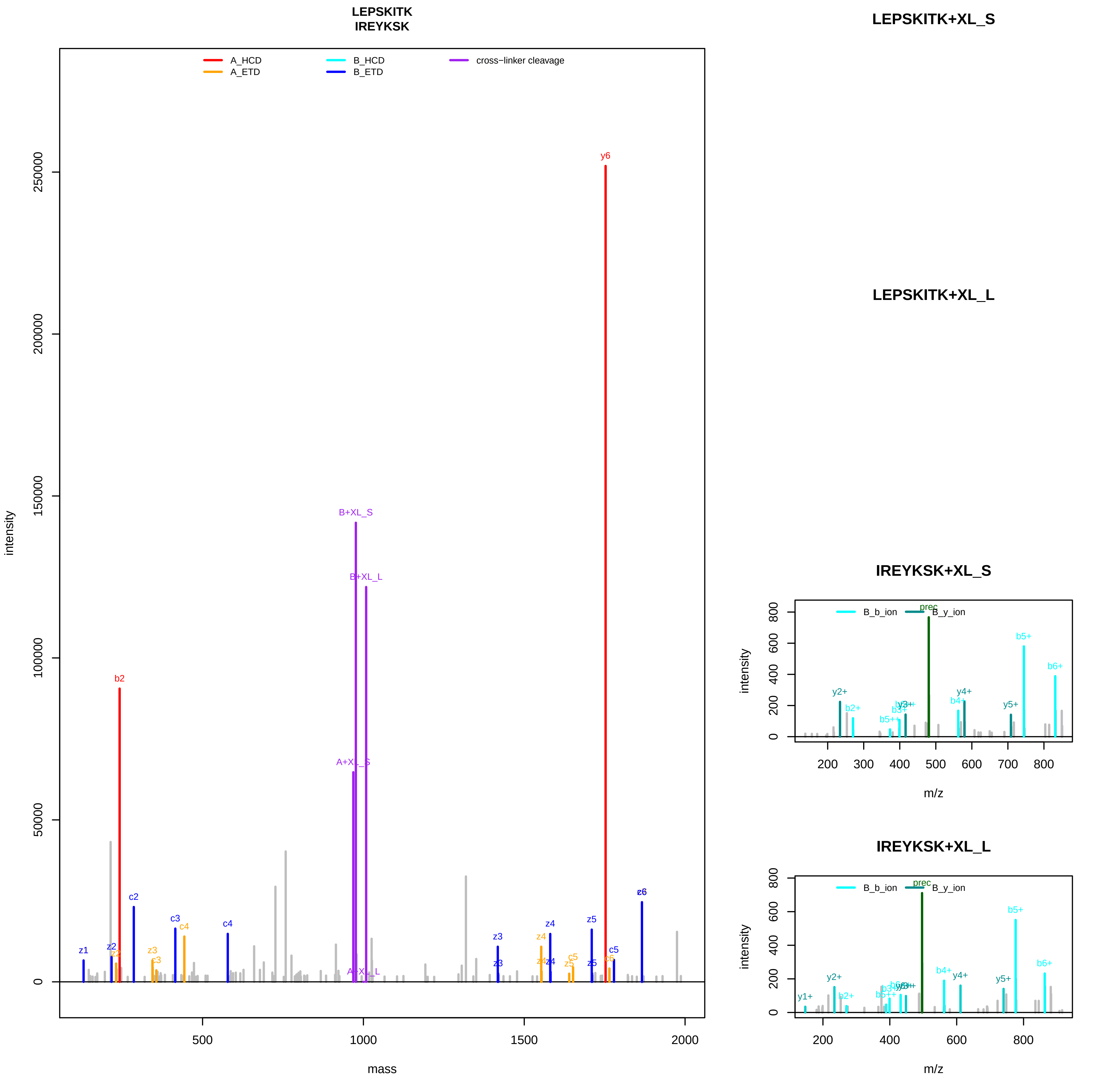
LEPSKITK
IREYKSK
A_HCD
A_ETD
B_HCD
B_ETD
cross−linker cleavage
intensity
mass
y6
b2
B+XL_S
B+XL_L
A+XL_S
A+XL_L
z1
z2
c2
c3
c4
z3
z4
z5
c5
c6
z6
0
50000
100000
150000
200000
250000
500
1000
1500
2000
LEPSKITK+XL_S
LEPSKITK+XL_L
IREYKSK+XL_S
IREYKSK+XL_L
B_b_ion
B_y_ion
prec
y1+
y2+
b2+
b3+
y3+
b4+
y4+
b5+
y5+
b6+
m/z
0
200
400
600
800
300
500
700
